# Latent Profiles of Decision Regret and Associated Factors Among Patients Undergoing Medium- and Long-Term Vascular Access Device Placement in Medical Oncology: A Cross-Sectional Study

**DOI:** 10.3390/curroncol33100612

**Published:** 2026-10-12

**Authors:** Xiaoyu Guo, Bo Zheng, Qiang Liu, Zhan Zhang, Yaping Zhu, Yanru Qiu, Yonghui Wan

**Affiliations:** 1 Department of Oncology, Renmin Hospital of Wuhan University, Wuhan 430060, China2025283070061@whu.edu.cn (Y.Z.); 2 Medical College, Yangtze University, Jingzhou 434023, China; 2024721143@yangtzeu.edu.cn; 3 Wuhan First Hospital, Wuhan 430022, China

**Keywords:** vascular access, decision regret, latent profile analysis, patients with cancer, shared decision-making, decisional conflict

## Abstract

**Background:** Patients receiving medical oncology care must weigh multiple factors when selecting a vascular access device, such as a peripherally inserted central catheter, implantable port, or midline catheter. Decision regret is an important patient-reported outcome that may adversely affect treatment adherence and quality of life. From the perspectives of shared decision-making and precision nursing, identifying subgroups of patients with distinct patterns of decision regret may help provide individualized decision support. **Objective:** To identify latent subgroups of decision regret among patients with cancer who underwent vascular access device placement, based on the three-dimensional structure of decision regret—process regret, option regret, and outcome regret—and to examine the associations of demographic characteristics, clinical factors, decisional conflict, and shared decision-making with subgroup membership. **Methods**: A cross-sectional survey was conducted using convenience sampling. A total of 309 patients who had undergone vascular access device placement and participated in treatment-related decision-making were recruited from the oncology department of a tertiary hospital in China between February and June 2026. Data were collected using a researcher-designed demographic and clinical characteristics questionnaire, the Three-Dimensional Decision Regret Scale, the Decisional Conflict Scale, and the Shared Decision-Making Questionnaire. Latent profile analysis was performed to identify subgroups of decision regret, and multinomial logistic regression was used to examine factors associated with subgroup membership. **Results:** A total of 309 patients were included in the final analysis. Based on model fit indices, LMR/BLRT results, entropy, class proportions, and clinical interpretability, the three-profile solution was selected and identified three latent profiles: low decision regret (77.35%), moderate decision regret (13.59%), and high decision regret (9.06%). Univariable analyses showed that place of residence, educational attainment, employment status, monthly household income, type of vascular access device, family involvement in decision-making, receipt of decision-related education, decisional conflict score, and shared decision-making score differed significantly across profiles (all *p* < 0.05). In the multinomial logistic regression model, place of residence, educational attainment, employment status, family involvement in decision-making, receipt of decision-related education, decisional conflict score, and shared decision-making score were associated with profile membership. After correcting for classification uncertainty using R3STEP, DCS and SDM-Q-9 remained significantly associated with profile membership, whereas the association with employment status was no longer significant. However, the high-regret group was small (*n* = 28), and the confidence intervals for several predictors were wide (e.g., unemployment: relative risk ratio (RRR) = 30.5, 95% CI 3.24–288). Monthly household income and type of vascular access device did not remain statistically significant. **Conclusions**: Substantial heterogeneity in decision regret was observed among patients with cancer who underwent vascular access device placement. Particular attention should be given to patients who experience high levels of decisional conflict or receive insufficient preprocedural education. Profile-based nursing interventions and enhanced shared decision-making before device placement are promising strategies, but their effects need to be evaluated in prospective studies.

## Simple Summary

Patients with cancer often need a special device placed in a vein to receive treatment safely. Several types of devices exist, each with different benefits and drawbacks. Some patients later feel they made the wrong choice—a feeling known as decision regret. We surveyed 309 patients in China who had recently received such a device. Most had little regret, but some had moderate or high regret. Those who felt more conflicted, were less involved in choosing, or received less information beforehand were more likely to feel regret. These findings suggest that health professionals should better involve patients in the decision and provide clearer information before the device is placed. This may reduce regret and improve the treatment experience.

## 1. Introduction

Cancer continues to impose a substantial global disease burden. In 2022, the number of newly diagnosed cancer cases remained high worldwide, with an emerging trend toward a younger age at diagnosis [1]. For most patients with cancer who require chemotherapy, targeted therapy, or long-term supportive care, safe, reliable, and guideline-concordant vascular access is fundamental to the successful delivery of anticancer treatment throughout the continuum of care [2]. Currently, a range of vascular access options is available for patients with cancer, including peripheral venous access, central venous catheters, and totally implantable venous access devices (TIVADs) [3]. However, substantial differences exist among these vascular access options with respect to complication risks, indwelling duration, maintenance requirements, restrictions on daily activities, and treatment-related costs [4]. Selection of a vascular access device requires patients to balance its long-term safety and appropriateness for the intended treatment with their individual lifestyle, physical condition, and financial circumstances [5]. Given the potential complications and long-term uncertainties associated with each vascular access option, as well as the invasive nature of device placement, patients may experience self-doubt, emotional distress, and repeated negative appraisal after making a decision. Decision regret has therefore become an important psychological concern in this population and may compromise treatment adherence and long-term quality of life [6].

Throughout the processes of vascular access decision-making and postprocedural psychological adjustment, healthcare professionals have the closest contact with patients and therefore play a pivotal role in providing information, emotional support, and ongoing care [7]. Healthcare professionals can provide patients with comprehensive, evidence-based information on the indications, advantages, and limitations of different vascular access options, thereby improving their understanding of the disease and treatment and reducing uninformed decisions caused by information asymmetry [8]. In addition, through effective communication and emotional support, healthcare professionals can encourage patients to express their preferences and values, thereby enhancing the overall decision-making experience [9]. With the continued advancement of patient-centered care and precision nursing, the value of shared decision-making (SDM) in oncology has become increasingly recognized. SDM has been shown to improve patients’ healthcare experiences, treatment satisfaction, and ability to adjust to illness [10]. Globally, approaches to medical decision-making vary substantially according to sociocultural norms, family structures, and prevailing models of care. In oncology, family members commonly participate in, assist with, or even assume a dominant role in treatment-related decisions. Although such involvement may provide valuable support, it may also limit patients’ opportunities to express their autonomous preferences, thereby increasing the risk of psychological distress and subsequent decision regret [11]. Existing evidence suggests that patients with cancer are increasingly willing to participate in healthcare decision-making. However, systematic research on shared decision-making practices, decisional conflict, and decision regret in the context of vascular access remains limited [12]. Most previous studies have focused on objective outcomes, such as vascular access-related complications and the effectiveness of nursing care, whereas the psychological heterogeneity of decision regret has not been adequately explored. In particular, subgroups characterized by distinct patterns of decision regret have not been systematically identified. Consequently, the psychological profiles of this population remain poorly understood, limiting the development of targeted interventions. Decision regret is a well-recognized adverse psychological outcome following healthcare decisions and is influenced by multiple factors, including individual decision-making capacity, access to information, social support, decisional conflict, and the quality of shared decision-making. Accordingly, substantial heterogeneity in decision regret may exist across patient populations [13]. Traditional variable-centered analyses based on overall mean scores are limited in their ability to distinguish subgroups with different psychological characteristics and may therefore fail to identify patients at high risk. Latent profile analysis (LPA), a person-centered statistical approach, can classify individuals into relatively homogeneous subgroups based on multiple psychological indicators, thereby addressing important limitations of conventional analytic methods [14]. Previous studies have shown that decisional conflict, the level of shared decision-making, and demographic and clinical characteristics are closely associated with patients’ psychological responses to healthcare decisions [15,16,17]. However, the extent to which these factors influence distinct profiles of decision regret among patients with cancer undergoing vascular access device placement remains unclear, and the available evidence is inconsistent [18]. Improving the psychological experience of decision-making and reducing decision regret are important goals of precision oncology nursing and high-quality healthcare. Accordingly, the present study used LPA to identify distinct profiles of decision regret among patients with cancer undergoing vascular access device placement. Differences in shared decision-making and decisional conflict across these profiles were examined, and the associations of demographic, clinical, and decision-related psychological factors with profile membership were further evaluated. The study aimed to characterize the heterogeneity and associated factors of decision regret in this population and to provide an empirical basis for the development of targeted psychological and decision-support interventions.

## 2. Materials and Methods

### 2.1. Study Population

Convenience sampling was used to recruit patients from a tertiary hospital in China between February and June 2026. The inclusion criteria were as follows: (1) age ≥ 18 years; (2) placement of a venous access device (VAD) for the administration of anticancer therapy, with an indwelling duration of ≤3 months; (3) awareness of and active participation in the vascular access decision-making process; and (4) adequate comprehension and communication abilities to complete the questionnaire or interview independently. The exclusion criteria were as follows: (1) marked deterioration in clinical condition or the presence of other severe physical illnesses that precluded participation; (2) unwillingness or inability to cooperate with the study procedures; (3) lack of awareness of the cancer diagnosis; and (4) cognitive impairment, psychiatric disorders, or other conditions that prevented completion of the questionnaire. The study was approved by the Ethics Committee of Renmin Hospital of Wuhan University (approval No. WDRY2026-K040). Written informed consent was obtained from all participants before data collection.

### 2.2. Study Procedure

The research team comprised five members: one principal investigator, two questionnaire administrators, one quality-control investigator, and one statistical analyst. Before data collection, all team members received standardized training covering the study protocol, interpretation of questionnaire items, standardized administration and completion procedures, and key principles of patient–clinician communication. Data were collected through one-to-one, face-to-face questionnaire surveys. Potential participants were screened strictly according to the predefined eligibility criteria. Eligible patients were provided with a detailed explanation of the study objectives, survey content, and questionnaire completion requirements. After written informed consent had been obtained, participants completed the questionnaires independently. Questionnaire completion required approximately 10–15 min. When clarification of an item was requested, a standardized explanation was provided by the investigator without influencing the participant’s response. Completed questionnaires were collected and reviewed immediately for missing responses, incomplete items, unclear answers, and patterned or otherwise invalid responses. Participants were asked to complete or clarify responses when necessary. Clinical information that could not be accurately recalled was verified against the hospital’s electronic medical records. All valid data were independently entered into the database by two investigators using a double-entry procedure. Discrepancies were identified and resolved to maximize the accuracy and reliability of the dataset before statistical analysis.

### 2.3. Assessment Tools

Demographic and Clinical Instrument. A researcher-developed demographic and clinical characteristics questionnaire was designed based on a review of relevant Chinese and international literature and consultation with experts. Demographic variables included age, place of residence, sex, marital status, educational attainment, employment status, monthly household income, and type of medical insurance. Clinical and decision-related variables included the type of vascular access device placed, family caregiver involvement in decision-making, and receipt of decision-related education or counseling. Family involvement in decision-making was defined as whether a family member or caregiver participated in the treatment-related decision-making process regarding vascular access device selection. It was measured using a single binary item (yes/no) in the researcher-developed demographic and clinical characteristics questionnaire, with the reference period being the decision-making process before device placement. Decision-related education was defined as whether the patient received any education or counseling about the available vascular access device options before placement. It was also measured using a single binary item (yes/no) in the same questionnaire, with the timing specified as before device placement.

The Decisional Conflict Scale (DCS) was originally developed by O’Connor at the University of Ottawa [19]. The Decisional Conflict Scale (DCS) is widely used to assess the degree of conflict experienced by patients during healthcare decision-making. It can be used to identify patients’ decision-support needs, evaluate the quality of the decision-making process, assess the effectiveness of decision-support interventions, and measure uncertainty regarding a course of action. Owing to its satisfactory reliability, validity, and clinical applicability, the DCS has been translated into multiple languages and widely used across countries. The DCS consists of 16 items, each rated on a 5-point Likert scale ranging from 0 (“strongly agree”) to 4 (“strongly disagree”). The total score is calculated by summing the item scores, dividing the sum by 16, and multiplying the result by 25, yielding a standardized score ranging from 0 to 100. Higher scores indicate greater decisional conflict. Scores below 25 are generally associated with decision implementation, whereas scores above 37.5 are associated with decision delay or uncertainty regarding the chosen option [20]. Cronbach’s α coefficients for the original subscales ranged from 0.78 to 0.92. In 2015, Lam et al. [21] translated and culturally adapted the DCS for use among Chinese populations in Hong Kong, reporting Cronbach’s α coefficients ranging from 0.51 to 0.87. In the present study, the DCS was administered after vascular access device placement (within 3 months post-placement). Accordingly, the DCS scores reflect the decisional conflict that patients were still experiencing at the time of assessment, rather than a retrospective recall of their feelings during the initial decision-making process. In the present sample, the Cronbach’s α coefficient was 0.964.

The Three-Dimensional Decision Regret Scale was developed by Van Tol-Geerdink et al. in 2016 [22], based on decision justification theory (DJT) [23]. The scale was translated and culturally adapted into Chinese by Wang et al. in 2025 [24]. The Chinese version demonstrated good internal consistency, with a Cronbach’s α coefficient of 0.913 for the total scale and coefficients ranging from 0.842 to 0.925 for the three subscales. The instrument contains 18 items rated on a 5-point Likert scale ranging from 1 (“strongly disagree”) to 5 (“strongly agree”). Items 1, 3, 4, 7, 8, 10, 13, 14, and 17 are reverse scored. Total scores range from 18 to 90, with higher scores indicating greater decision regret. In the present sample, the Cronbach’s α coefficients were 0.931 for the total scale and 0.780, 0.931, and 0.934 for the process (7 items; range 7–35), option (7 items; range 7–35), and outcome (4 items; range 4–20) regret subscales, respectively.

The 9-item Shared Decision-Making Questionnaire (SDM-Q-9) was developed by Kriston et al. in 2010 [25] as a generic instrument for assessing patients’ perceptions of collaboration with healthcare professionals during medical decision-making. The questionnaire consists of nine items rated on a 6-point Likert scale ranging from 0 (“completely disagree”) to 5 (“completely agree”), yielding a total score of 0–45. Higher scores indicate a greater perceived level of patient–clinician collaboration during decision-making. The Chinese version was translated and validated by Luo et al. [26]. It demonstrated excellent internal consistency, with a Cronbach’s α coefficient of 0.945, and its validity indices met accepted psychometric criteria, supporting its use in assessing patients’ perceived participation in shared decision-making. In the present sample, the Cronbach’s α coefficient was 0.963.

### 2.4. Statistical Methods

A database was established using EpiData version 3.0 (EpiData Association, Odense, Denmark), and all statistical analyses were performed using SPSS version 27.0 (IBM Corp., Armonk, NY, USA) and Mplus version 8.3 (Muthén & Muthén, Los Angeles, CA, USA). Continuous variables with an approximately normal distribution were expressed as the mean ± standard deviation (SD), whereas categorical variables were summarized as frequencies and percentages. Differences in nominal categorical variables among groups were assessed using the chi-square test or Fisher’s exact test, as appropriate. Comparisons of ordinal variables across multiple groups were performed using the Kruskal–Wallis H test. A two-sided *p* value < 0.05 was considered statistically significant. Latent profile analysis (LPA) was performed in Mplus 8.3 using the MLR estimator. The three subscale scores of the Three-Dimensional Decision Regret Scale—process regret, option regret, and outcome regret—were entered as continuous indicators. Variances were constrained to be equal across classes, and covariances among indicators were fixed to zero. Random starts were set at 500 initial-stage and 100 final-stage optimizations. All models terminated normally, and the best log-likelihood value was replicated. Model fit was evaluated using the Akaike information criterion (AIC), Bayesian information criterion (BIC), sample-size-adjusted Bayesian information criterion (aBIC), entropy, the Lo-Mendell-Rubin adjusted likelihood ratio test (LMR-aLRT), and the bootstrap likelihood ratio test (BLRT). Lower AIC, BIC, and aBIC values indicated better model fit. Entropy values closer to 1 indicated greater classification accuracy, with values > 0.80 considered indicative of good classification quality. Statistically significant LMR-aLRT and BLRT results (*p* < 0.05) indicated that the model with *k* latent profiles provided a significantly better fit than the model with *k* − 1 profiles. Latent profile membership was subsequently entered as the dependent variable in regression analyses. Variables that were statistically significant in the univariable analyses were entered into the multinomial logistic regression model as independent variables. Because the proportional odds assumption was violated, ordinal logistic regression was considered inappropriate; therefore, multinomial logistic regression was performed to estimate the associations between the candidate variables and latent profile membership. A parsimonious sensitivity analysis including only the three core variables (DCS score, SDM-Q-9 score, and employment status) was conducted to explore the robustness of the main findings. These variables were selected because they showed the strongest and most consistent associations in the main model and were the most theoretically relevant predictors of decision regret. The results are presented in Supplementary Table S1. Average marginal effects (AMEs) were calculated for the key predictors to provide absolute effect sizes (Supplementary Table S2). To account for classification uncertainty in latent profile assignment, a corrected three-step covariate analysis (R3STEP) was conducted in Mplus. This approach estimates the associations between covariates and latent profile membership while adjusting for classification error. The results are presented in Supplementary Table S3. To address overfitting and quasi-separation, Firth penalized logistic regression was performed for the two binary contrasts (Class 2 vs. Class 1 and Class 3 vs. Class 1), with five core predictors (DCS, SDM-Q-9, family involvement, education, and unemployment). The results are presented in Supplementary Table S4. Given the exploratory and hypothesis-generating nature of this study, no formal adjustment for multiple comparisons was applied. However, this may increase the risk of Type I error, and the results should be interpreted accordingly.

### 2.5. Sample Size

Based on methodological recommendations, a total sample of at least 300 participants is required to obtain stable latent profile solutions [27], and each identified class should ideally account for no less than 5% of the total sample to ensure stable parameter estimation [28]. A total of 309 valid questionnaires were included in the final analysis, meeting the recommended sample size threshold for LPA.

## 3. Results

### 3.1. Demographic and Clinical Characteristics of the Participants

A total of 320 questionnaires were distributed, and 309 valid questionnaires were returned, yielding an effective response rate of 96.6%. The remaining 11 questionnaires were excluded because they were invalid, as defined by the prespecified quality-control criteria, including incomplete responses, patterned or inconsistent answers, or missing data on key variables. The demographic and clinical characteristics of the participants are shown in Table 1.

### 3.2. Latent Profiles of Decision Regret

Although the four-profile model showed lower AIC and BIC values and a significant BLRT, the LMR-aLRT for 4 versus 3 profiles was not significant. Inspection of the four-profile means revealed that two classes had very similar mean scores across all three regret dimensions, suggesting that the four-profile solution may have split the low-regret group into two clinically indistinguishable subgroups. Given the exploratory aim and the need for parsimonious, interpretable profiles, we retained the three-profile solution. We acknowledge that information criteria alone favored the four-profile solution, and this decision should be interpreted with caution. The model fit indices for the latent profile solutions are presented in Table 2.

**Figure 1:**
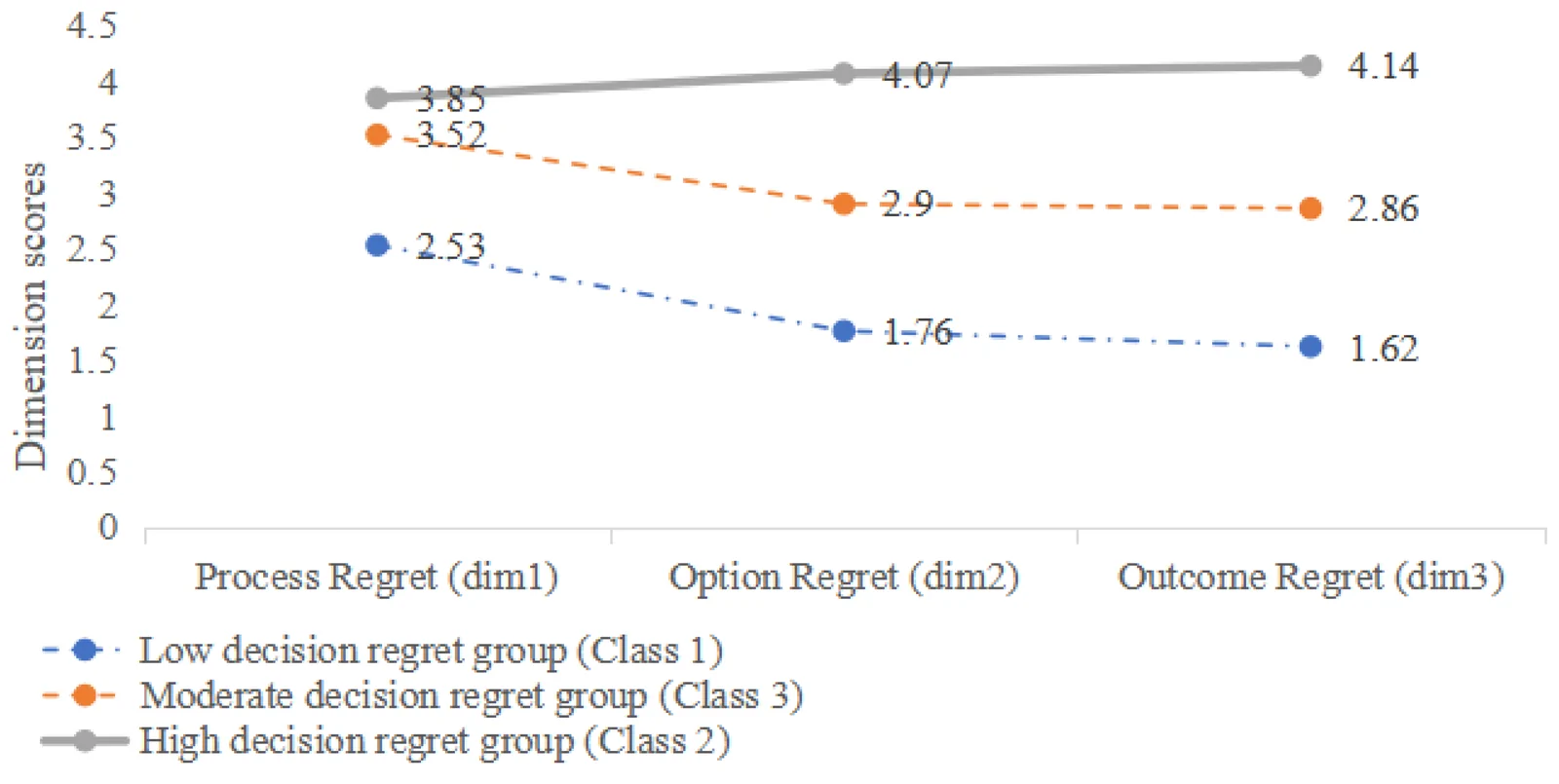
Three-category latent profile analysis of decision regret for vascular access selection. Scores are presented as item means (subscale total divided by the number of items) to facilitate comparison across subscales with different ranges.

Model convergence was satisfactory. All models terminated normally, and the best log-likelihood value was replicated across multiple random starts. For the three-profile solution, the entropy value was 0.930, indicating excellent classification accuracy. The average posterior probabilities for the most likely class membership were 0.986, 0.911, and 0.915 for Classes 1, 2, and 3, respectively, further supporting the stability of the three-profile solution. It should be noted that entropy reflects the quality of classification (i.e., how accurately individuals are assigned to their most likely class) rather than the stability or superiority of the selected solution. Model selection was based on multiple criteria, including information criteria, likelihood ratio tests, class proportions, and interpretability. The three latent profiles of decision regret across the three dimensions are shown in Figure 1.

The score differences across the three dimensions among the three latent profiles are shown in Table 3.

Univariate analyses showed statistically significant differences among the three latent profile groups in place of residence, educational attainment, employment status, monthly household income, type of vascular access device, family involvement in decision-making, receipt of decision-related education, DCS score, and SDM-Q-9 score (all *p* < 0.05). No statistically significant differences were observed among the three groups in age, sex, marital status, or type of medical insurance (all *p* > 0.05). The results of the univariate analysis are shown in Table 4.

The variables that were statistically significant in the univariable analyses were entered into a multinomial logistic regression model, with the three decision-regret profiles treated as the dependent variable. Compared with Class 1 (low decision regret), rural residence, completion of senior high school or technical secondary school, receipt of decision-related education, and a higher Shared Decision-Making Questionnaire (SDM-Q-9) score were associated with lower odds of membership in Class 2 (high decision regret). In contrast, being unemployed, having lost or left a job, and a higher Decisional Conflict Scale (DCS) score were associated with higher odds of Class 2 membership (all *p* < 0.05). Compared with Class 1, family involvement in decision-making, receipt of decision-related education, and a higher SDM-Q-9 score were associated with lower odds of membership in Class 3 (moderate decision regret), whereas being unemployed, having lost or left a job, and a higher DCS score were associated with higher odds of Class 3 membership (all *p* < 0.05). No variables were significantly associated with membership in Class 2 vs. Class 3 (all *p* > 0.05). In the AME analysis, a 10-point increase in DCS score was associated with a 5.3 percentage point increase in the probability of high regret membership (AME = 0.053, *p* = 0.018), whereas a 10-point increase in SDM-Q-9 score was associated with a 4.2 percentage point decrease (AME = −0.042, *p* = 0.001) (Supplementary Table S2). In the R3STEP analysis, the associations of DCS and SDM-Q-9 with profile membership remained consistent with the main model (Class 2 vs. Class 1: DCS RRR = 1.304, *p* = 0.040; SDM RRR = 0.640, *p* = 0.007; Class 3 vs. Class 1: DCS RRR = 1.151, *p* = 0.004; SDM RRR = 0.880, *p* = 0.002), supporting the robustness of these findings. Some categorical variables (e.g., unemployment, income) could not be reliably estimated due to quasi-separation and the small size of the high-regret group (*n* = 28). The full results are provided in Supplementary Table S3. In the Firth penalized regression, DCS and SDM-Q-9 remained significantly associated with both high and moderate regret in the expected direction (Class 2 vs. Class 1: DCS OR = 1.161, *p* = 0.002; SDM OR = 0.892, *p* = 0.021; Class 3 vs. Class 1: DCS OR = 1.134, *p* < 0.001; SDM OR = 0.921, *p* = 0.012). Family involvement was significantly associated with lower odds of moderate regret (OR = 0.276, *p* = 0.007) but not high regret. Unemployment was not significantly associated with either contrast after penalization, consistent with the R3STEP analysis. The results of the multinomial logistic regression analysis are shown in Table 5.

## 4. Discussion

To our knowledge, this is the first study to use latent profile analysis (LPA) to identify distinct profiles of decision regret related to vascular access device selection among patients with cancer. Three latent profiles were identified: low, moderate, and high decision regret. These findings extend the current understanding of decision regret in vascular access care by demonstrating substantial heterogeneity within this population and provide an empirical basis for the development of profile-specific decision-support interventions.

The low-regret profile accounted for the largest proportion of the sample, indicating that most participants experienced relatively low levels of regret following vascular access device placement. Nevertheless, clinically meaningful subgroups with moderate and high decision regret were also identified, suggesting that post-decision regret remains an important concern for a substantial proportion of patients. The three profiles showed a clear graded pattern across all dimensions of decision regret: scores for process regret, option regret, and outcome regret increased progressively from the low-regret profile to the moderate-regret profile and then to the high-regret profile. This consistent gradient supports the clinical interpretability of the identified profiles and suggests that the profiles reflected broad differences in the overall intensity of decision regret rather than isolated differences in a single dimension. Selection of a vascular access device requires patients to weigh multiple factors, including potential complications, duration of use, maintenance requirements, restrictions on daily activities, treatment compatibility, and financial burden. At the time of assessment, patients who had experienced complications, inconvenience, or unexpected treatment experiences may report greater regret regarding their original choice. Most previous studies have examined decision regret using total scale scores and variable-centered analyses. Although such approaches are useful for estimating overall regret, they may obscure clinically meaningful heterogeneity in patients’ experiences. The present person-centered analysis therefore provides additional insight into subgroups that may require different forms and intensities of decision support. Decision-making practices may also be influenced by sociocultural and healthcare contexts. In some Western healthcare settings, patients may be more likely to receive structured preprocedural education and formal shared decision-making support, which may help them establish clearer expectations regarding the benefits, limitations, and likely outcomes of different vascular access options [29]. In China, medical decisions are often made with substantial involvement from clinicians and family members. Although such involvement may provide emotional and practical support, excessive delegation of decision-making authority may reduce patients’ sense of participation in or ownership of the final choice. When complications or other unfavorable outcomes occur, reduced personal involvement may contribute to subsequent decision regret [30]. These cross-cultural interpretations should, however, be considered cautiously because decision-making preferences vary considerably both within and across healthcare systems.

Pairwise comparisons showed that statistically significant factors were identified when the low-regret profile was compared with either the moderate- or high-regret profile.

In contrast, no statistically significant variables distinguished the high-regret profile from the moderate-regret profile. However, this absence of significant differences should not be interpreted as evidence that the two groups are similar. Given the small size of the high-regret group (*n* = 28) and the resulting low statistical power, the confidence intervals for several predictors were wide, and meaningful differences between the two groups cannot be excluded. Therefore, the lack of significant contrasts may reflect limited statistical power rather than a true absence of differences. Future studies with larger samples are needed to determine whether the moderate- and high-regret profiles differ in the nature, rather than merely the intensity, of their associated factors.

Higher decisional conflict was associated with greater odds of membership in both the moderate- and high-regret profiles relative to the low-regret profile. Patients with substantial decisional conflict may have insufficient information, unresolved uncertainty, competing preferences, or difficulty weighing the advantages and disadvantages of available vascular access options. These unresolved concerns may weaken confidence in the selected option and make patients more likely to question their choice after device placement. It is important to note that the DCS was administered after device placement, at the same time as the regret scale. Residual decisional conflict after the decision may overlap conceptually with regret, particularly in the uncertainty and effective-decision subscales. Therefore, the strong association between DCS and regret may partly reflect common-method variance or conceptual overlap, rather than a purely causal relationship. Future studies should measure decisional conflict before the decision and regret at a later time point to disentangle these constructs. The findings therefore support routine assessment of decisional conflict before vascular access decisions are finalized. Patients with elevated DCS scores may benefit from additional information, values clarification, individualized counseling, and opportunities to reconsider the available options before placement. Higher levels of shared decision-making were associated with lower odds of membership in both the moderate- and high-regret profiles. Shared decision-making may be associated with lower regret by improving patients’ understanding of the available options, facilitating the expression of their preferences and values, and strengthening their sense of involvement in the final decision. Even when complications or unfavorable outcomes subsequently occur, a decision that is consistent with the patient’s informed preferences may remain more psychologically acceptable. These findings suggest that shared decision-making should be regarded not only as a communication strategy but also as an important component of psychological support in vascular access care.

Receipt of decision-related education was also associated with lower odds of membership in both the moderate- and high-regret profiles. Structured preprocedural education may improve patients’ understanding of the indications, benefits, limitations, maintenance requirements, and potential complications of each vascular access option. It may also help patients establish realistic expectations and reduce discrepancies between anticipated and actual experiences. Decision-related education should therefore extend beyond technical explanations and include comparisons among devices, likely effects on daily life, maintenance responsibilities, possible complications, and opportunities for patients to raise individual concerns. Employment status was another important factor. Patients who were unemployed or not currently working had higher odds of belonging to both the moderate- and high-regret profiles. This association may partly reflect the persistent financial and psychological burden associated with cancer treatment and the continuing costs of vascular access device maintenance. However, although household income differed significantly across the three profiles in the univariable analysis, it did not remain statistically significant in the multivariable model. The association between employment status and decision regret may therefore not be explained solely by financial strain. Employment status may also reflect social participation, role stability, perceived control, and psychological security. Further research is needed to clarify the mechanisms underlying this association. However, in the R3STEP analysis accounting for classification uncertainty, the association between unemployment and regret was no longer statistically significant (Class 2 vs. Class 1: *p* = 0.739; Class 3 vs. Class 1: *p* = 0.268), likely due to the small size of the high-regret group and quasi-separation. In the parsimonious sensitivity analysis, the associations of DCS and SDM-Q-9 with profile membership remained consistent with the main model, supporting the robustness of these two predictors. However, the association between unemployment and high regret was attenuated (RRR decreased from 30.55 to 4.09), and its association with moderate regret became nonsignificant (*p* = 0.079). These findings indicate that the employment-related results are less stable and should be interpreted with caution. The confidence intervals for unemployment were extremely wide (RRR = 30.5, 95% CI 3.24–288), which likely reflects quasi-separation due to the small size of the high-regret group. Family involvement in decision-making was significantly associated with lower odds of membership in the moderate-regret profile relative to the low-regret profile (Class 3 vs. Class 1: RRR = 0.333, *p* = 0.036), but not with high regret (Class 2 vs. Class 1: *p* = 0.441). Appropriate family participation may provide emotional reassurance, practical assistance, and support in understanding complex medical information. Nevertheless, family involvement should complement rather than replace the patient’s own preferences and values. Healthcare professionals should assess the patient’s desired level of family participation and ensure that the final decision remains consistent with the patient’s informed preferences. In univariable analyses, rural residence and educational attainment were significantly associated with profile membership. After adjustment for other variables, rural residence (RRR = 0.194, *p* = 0.025) and senior high school education (RRR = 0.021, *p* = 0.033) remained significantly associated with lower odds of high regret (Class 2 vs. Class 1). However, these associations were not statistically significant for moderate regret (Class 3 vs. Class 1: rural residence, RRR = 0.433, *p* = 0.194; senior high school, RRR = 0.544, *p* = 0.581). Therefore, their effects on moderate regret should be interpreted with caution, while their associations with high regret appear more robust. It is possible that other unmeasured factors—such as health literacy, expectations regarding participation, or preferences for delegating decisions—may contribute to these associations, but these cannot be determined from the present data. Demographic characteristics should not be used in isolation to classify patients as being at low risk. Although household income and vascular access device type differed significantly across the profiles in the univariable analyses, neither remained statistically significant in the multivariable model. Their apparent associations may therefore be explained, at least partly, by other demographic, clinical, or psychological factors included in the model. However, because data on device-related complications (e.g., infection, occlusion, thrombosis) were not collected, we could not adjust for these potential confounders or mediators. The univariate association between device type and decision regret may therefore be confounded by complication risk, and no independent effect of device type on regret can be established from the present data. Larger multicenter studies are needed to clarify whether these variables have effects in more diverse populations. Taken together, these findings highlight the importance of combining demographic screening with psychological and behavioral assessment. Decisional conflict and shared decision-making remained associated with profile membership after demographic and clinical characteristics were considered. Clinical risk assessment should therefore incorporate DCS scores, patients’ perceived participation in decision-making, decision-support needs, and receipt of preprocedural education. Such a multidimensional approach may identify patients who would benefit from targeted decision support more accurately than demographic screening alone.

The high-regret subgroup should not be interpreted as evidence of a spurious class resulting from model overfitting, despite its small proportion. Although some person-centered studies have reported that favorable outcomes may represent a minority [31], our findings showed that the low-regret profile was the largest subgroup (77.35%). This discrepancy may reflect differences in study populations and outcome measures. Provided that the model demonstrates satisfactory fit, adequate entropy, acceptable posterior classification probabilities, and meaningful between-profile differences validated by external covariates, a small subgroup may still have substantial clinical relevance and should not be dismissed solely because of its limited size [32]. Because the present study used a cross-sectional design, we cannot determine whether low regret persisted over time; future longitudinal studies are needed to examine changes in decision regret over time, consistent with previous trajectory-based research in oncology populations [33].

## 5. Limitations

This study has several limitations. First, the small size of the high-regret profile (*n* = 28) may have led to quasi-separation and small-cell instability in the multinomial logistic regression, as reflected by the extremely wide confidence intervals for several predictors (e.g., unemployment: RRR = 30.5, 95% CI 3.24–288). This limits the precision of our effect estimates and may have reduced statistical power. Firth penalized regression was performed to address small-sample bias; however, the high-regret group remained small, and the results should be interpreted with caution. In the R3STEP analysis accounting for classification uncertainty, the association between unemployment and regret was no longer significant (Class 2 vs. Class 1: *p* = 0.739; Class 3 vs. Class 1: *p* = 0.268), whereas DCS and SDM-Q-9 remained significantly associated with profile membership. Second, the cross-sectional design and single time-point measurement of regret preclude causal inference and cannot capture changes in regret over time; retrospective self-report may also be subject to recall bias. In addition, the DCS and regret scale were administered at the same time point, which may have inflated their association due to common-method variance and conceptual overlap. Third, data on vascular access-related complications (e.g., infection, thrombosis, occlusion) were not collected, because the present study focused on patient-reported psychological outcomes and decision-making processes rather than clinical outcomes. These complications are potential confounders that may influence patients’ evaluation of the device and subsequent regret. Future studies should integrate clinical complication data with psychological assessments. Fourth, participants were recruited from a single tertiary hospital in China, limiting generalizability to other settings and populations. Fifth, patient-reported data may be affected by social desirability and recall biases. Finally, other potentially important factors—such as patient–clinician communication quality, health literacy, and reasons for device selection—were not assessed. Larger, multicenter, longitudinal studies are needed to confirm and extend these findings. In addition, the exact time since device placement, cancer stage, and treatment intent were not systematically collected. Regret is time dependent, and disease stage and treatment intent may influence device selection and subsequent regret. Future studies should record and adjust for these variables. Furthermore, no adjustment for multiple comparisons was applied, which may increase the risk of Type I error. In addition, the demographic and clinical questionnaire was researcher-designed and did not undergo formal psychometric validation, which may have introduced measurement error. Furthermore, data were collected face-to-face by the recruiting team, and social desirability bias may have led patients to underreport decision regret, particularly when responding to the team involved in their care.

## 6. Conclusions

This study identified three distinct latent profiles of decision regret related to vascular access device selection among patients receiving medical oncology care: low, moderate, and high decision regret, indicating substantial heterogeneity within this population. Greater decisional conflict was associated with higher odds of membership in the moderate- and high-regret profiles, and this association remained robust after correcting for classification uncertainty. Unemployment was associated with higher odds in the main analysis, but this association was no longer significant in the R3STEP analysis. Receipt of decision-related education and higher levels of shared decision-making were associated with lower odds of membership in these profiles. However, given the small size of the high-regret group (*n* = 28) and the wide confidence intervals for several predictors, larger multicenter studies are needed to confirm these findings. In univariable analyses, rural residence and educational attainment were associated with profile membership. After correcting for classification uncertainty using R3STEP, DCS and SDM-Q-9 remained significantly associated with profile membership, whereas the association with employment status was no longer significant. After adjustment, rural residence and senior high school education remained significantly associated with lower odds of high regret (Class 2 vs. Class 1), but not with moderate regret (Class 3 vs. Class 1). These findings may facilitate the identification of patients at increased risk of decision regret. Clinical assessment should integrate demographic characteristics with decisional conflict and shared decision-making measures. Structured, individualized preprocedural education and meaningful patient involvement in vascular access decisions may be associated with better decision-making experiences and lower regret; however, causal relationships cannot be established from this cross-sectional study.

## Figures and Tables

**Table 1:** Demographic and clinical characteristics of participants (*n* = 309).

Variable	*n* (%)	Variable	*n* (%)
Age (years)	60.72 ± 12.12	Self-pay	11 (3.6)
Sex		Type of vascular access device	
Male	170 (55.0)	PICC	128 (41.4)
Female	139 (45.0)	Implantable venous access port	165 (53.4)
Residence		Midline catheter	16 (5.2)
Rural	147 (47.6)	Cancer diagnosis	
Urban	162 (52.4)	Gastrointestinal	120 (38.8)
Marital status		Lung	95 (30.7)
Married	294 (95.1)	Breast	25 (8.1)
Unmarried/divorced/widowed	15 (4.9)	Gynecological	19 (6.1)
Educational level		Head and neck	19 (6.1)
Junior high school or below	167 (54.0)	Lymphoma	15 (4.9)
Senior high school or technical secondary school	87 (28.2)	Other	16 (5.2)
Junior college	30 (9.7)	Treatment characteristics	
Bachelor’s degree or above	25 (8.1)	Chemotherapy alone	195 (63.1)
Employment status		Radiotherapy alone	44 (14.2)
Employed	95 (30.7)	Immunotherapy alone	31 (10.0)
Unemployed or not currently working	114 (36.9)	Targeted therapy alone	7 (2.3)
Retired	100 (32.4)	Combined therapy	25 (8.1)
Monthly household income (CNY)		Other	7 (2.3)
<3000	115 (37.2)	Family involvement in decision-making	
3000–5000	105 (34.0)	Yes	233 (75.4)
5000–8000	61 (19.7)	No	76 (24.6)
>8000	28 (9.1)	Received decision-related education	
Health insurance		Yes	203 (65.7)
Urban Employee Basic Medical Insurance	77 (24.9)	No	106 (34.3)
Urban and Rural Resident Basic Medical Insurance	55 (17.8)	DCS score (decisional conflict)	19.97 ± 10.64
Hubei Provincial Basic Medical Insurance	166 (53.7)	SDM-Q-9 score (shared decision-making)	28.36 ± 10.19

Note: Data are presented as *n* (%) or mean ± SD. DCS = Decisional Conflict Scale; SDM-Q-9 = 9-item Shared Decision Making Questionnaire; PICC = peripherally inserted central catheter. The DCS consists of 16 items rated 0–4, with a raw total score range of 0–64 (standardized to 0–100). The SDM-Q-9 consists of 9 items rated 0–5, with a total score range of 0–45. The Three-Dimensional Decision Regret Scale consists of 18 items rated 1–5, with a total score range of 18–90; subscale ranges are 7–35 for process regret, 7–35 for option regret, and 4–20 for outcome regret.

**Table 2:** Fitting indices of the latent profile model for decision regret regarding vascular access selection.

Model	AIC	BIC	aBIC	Entropy	LMR-aLRT *p*	BLRT *p*	Class Probability
Class1	5593.325	5615.725	5596.696				1
Class2	5124.929	5162.262	5130.546	0.96	<0.001	<0.001	0.820, 0.180
Class3	5047.506	5099.773	5055.371	0.93	0.0245	<0.001	0.772, 0.085, 0.143
Class4	4913.608	4980.808	4923.719	0.959	0.0722	<0.001	0.421, 0.113, 0.064, 0.402

Note: Model-estimated class proportions were 8.5% and 14.3% for the high- and moderate-regret profiles, whereas most likely class membership yielded 9.06% and 13.59%, respectively; this small difference reflects the distinction between estimated model proportions and actual classification counts. AIC = Akaike information criterion; BIC = Bayesian information criterion; aBIC = sample-size-adjusted Bayesian information criterion; LMR-aLRT = Lo-Mendell-Rubin adjusted likelihood ratio test; BLRT = bootstrap likelihood ratio test; *p* = *p*-value.

**Table 3:** Score differences of relevant items and total scores of the three dimensions for the evaluation of vascular access decision regret ($\bar{x}$ ± s).

Dimension	Low Decision Regret Group (Class1, *n* = 239)	Moderate Decision Regret Group (Class3, *n* = 42)	High Decision Regret Group (Class2, *n* = 28)	F	*p*
Process Regret (dim1)	17.70 ± 4.30	24.67 ± 3.12	26.96 ± 5.37	94.676	<0.01
Option Regret (dim2)	12.33 ± 2.73	20.29 ± 3.14	28.46 ± 3.96	467.974	<0.01
Outcome Regret (dim3)	6.49 ± 1.94	11.43 ± 2.06	16.57 ± 2.59	378.102	<0.01

**Table 4:** Univariate analysis for latent profiles of decision regret regarding vascular access selection among tumor patients (*n* = 309).

Variable	Total	Class 1	Class 2	Class 3	H/χ^2^ Value	*p*
Age	—	60.75 ± 12.37	57.57 ± 13.75	62.62 ± 8.90	H = 2.396	0.302
Residence					χ^2^ = 10.374	0.006 *
Rural	147	102 (69.4)	19	26		
Urban	162	137 (84.7)	9	16		
Sex					χ^2^ = 3.644	0.162
Female	139	106 (76.3)	17 (12.2)	16 (11.5)		
Male	170	133 (78.2)	11 (6.5)	26 (15.3)		
Marital Status					χ^2^ = 0.351	0.839
Married	294	228 (77.6)	26 (8.8)	40 (13.6)		
Unmarried	15	11 (73.3)	2 (13.3)	2 (13.3)		
Educational Level					H = 6.211	0.045 *
Junior High School or Below	167	119 (71.3)	20 (12.0)	28 (16.8)		
Senior High School	87	75 (86.2)	3 (3.4)	9 (10.3)		
Junior College	30	24 (80.0)	3 (10.0)	3 (10.0)		
Bachelor’s Degree or Above	25	21 (84.0)	2 (8.0)	2 (8.0)		
Employment Status					χ^2^ = 18.143	<0.01 *
Employed	95	82 (86.3)	2 (2.1)	11 (11.6)		
Unemployed	114	75 (65.8)	19 (16.7)	20 (17.5)		
Retired	100	82 (82.0)	7 (7.0)	11 (11.0)		
Income					H = 6.669	0.036 *
<3000 RMB	115	80 (69.6)	18 (15.7)	17 (14.8)		
3000–5000 RMB	105	88 (83.8)	4 (3.8)	13 (12.4)		
5000–8000 RMB	61	48 (78.7)	5 (8.2)	8 (13.1)		
>8000 RMB	28	23 (82.1)	1 (3.6)	4 (14.3)		
Type of Vascular Access Device					χ^2^ = 9.888	0.042 *
PICC	128	93 (72.7)	15 (11.7)	20 (15.6)		
Implanted Venous Port	165	136 (82.4)	9 (5.5)	20 (12.1)		
Midline Catheter	16	10 (62.5)	4 (25.0)	2 (12.5)		
Health Insurance Type					χ^2^ = 10.975	0.089
Urban Employee Basic Medical Insurance	77	66 (85.7)	4 (5.2)	7 (9.1)		
Urban and Rural Resident Basic Medical Insurance	55	41 (74.5)	2 (3.6)	12 (21.8)		
Hubei Provincial Medical Insurance	166	125 (75.3)	20 (12.0)	21 (12.7)		
Self-pay	11	7 (63.6)	2 (18.2)	2 (18.2)		
Caregiver Involvement in Decision-making					χ^2^ = 28.167	<0.001 *
Yes	233	197 (84.5)	15 (6.4)	21 (9.0)		
No	76	42 (55.3)	13 (17.1)	21 (27.6)		
Received Decision-related Education					χ^2^ = 79.75	<0.001 *
Yes	203	188 (92.6)	4 (2.0)	11 (5.4)		
No	106	51 (48.1)	24 (22.6)	31 (29.2)		
DCS	—	16.26 ± 7.30	37.57 ± 10.20	29.36 ± 9.563	H = 111.906	<0.001 *
SDM-Q-9	—	31.96 ± 7.42	12.07 ± 7.74	18.71 ± 8.356	H = 109.737	<0.001 *

Note: H = Kruskal–Wallis H test; χ^2^ = chi-square test; SD = standard deviation,* *p* < 0.05.

**Table 5:** Associated factors of patients’ decision regret regarding vascular access selection (*n* = 309). Average marginal effects are provided in Supplementary Table S2.

		Class 2 vs. Class 1				Class 3 vs. Class 1				Class 2 vs. Class 3		
Variable												
	B	RRR	95% CI	*p*	B	RRR	95% CI	*p*	B	RRR	95% CI	*p*
Residence												
Rural	−1.639	0.194	0.0464–0.815	0.025	−0.838	0.433	0.122–1.532	0.194	−0.800	0.449	0.068–2.980	0.407
Urban	—	—	—	—	—	—	—	—	—	—	—	—
Educational Level												
Junior High School or Below	−2.976	0.051	0.002–1.359	0.076	−0.24	0.787	0.080–7.702	0.837	−2.736	0.065	0.003–1.456	0.085
Senior High School	−3.846	0.021	0.001–0.728	0.033	−0.61	0.544	0.063–4.726	0.581	−3.236	0.039	0.001–1.151	0.060
Junior College	−1.134	0.322	0.011–9.618	0.513	−0.432	0.649	0.052–8.158	0.738	−0.702	0.496	0.019–13.266	0.676
Bachelor’s Degree or Above	—	—	—	—	—	—	—	—	—	—	—	—
Employment Status												
Employed	0.947	2.579	0.235–28.350	0.439	1.117	3.057	0.827–11.302	0.094	−0.17	0.844	0.088–8.083	0.883
Unemployed	3.419	30.546	3.236–288.306	0.003	1.582	4.867	1.255–18.874	0.022	1.837	6.276	0.823–47.882	0.076
Retired	—	—	—	—	—	—	—	—	—	—	—	—
Income												
<3000 RMB	0.585	1.795	0.074–43.455	0.719	0.01	1.010	0.135–7.584	0.992	0.575	1.776	0.101–31.306	0.695
3000~5000 RMB	−0.803	0.448	0.017–12.141	0.633	−0.374	0.688	0.099–4.789	0.706	−0.429	0.651	0.031–13.584	0.782
5000~8000 RMB	0.311	1.364	0.056–33.170	0.849	0.349	1.418	0.206–9.772	0.723	−0.039	0.962	0.050–18.432	0.980
>8000 RMB	—	—	—	—	—	—	—	—	—	—	—	—
Type of Vascular Access Device												
PICC	−0.269	0.764	0.041–14.261	0.857	1.050	2.857	0.227–35.899	0.416	−1.319	0.267	0.021–3.410	0.310
Implanted Venous Port	−1.975	0.139	0.007–2.945	0.205	−0.069	0.933	0.073–11.903	0.958	−1.907	0.149	0.011–2.077	0.157
Midline Catheter	—	—	—	—	—	—	—	—	—	—	—	—
Caregiver Involvement in Decision-making												
Yes	−0.596	0.551	0.121–2.511	0.441	−1.099	0.333	0.119–0.931	0.036	0.503	1.653	0.458–5.966	0.443
No	—	—	—	—	—	—	—	—	—	—	—	—
Received Decision-related Education												
Yes	−2.319	0.098	0.017–0.570	0.010	−1.964	0.140	0.049–0.404	<0.001	−0.356	0.701	0.135–3.640	0.672
No	—	—	—	—	—	—	—	—	—	—	—	—
DCS	0.190	1.209	1.092–1.340	<0.001	0.120	1.128	1.043–1.220	0.003	0.070	1.072	0.990–1.161	0.086
SDM-Q-9	−0.174	0.840	0.746–0.946	0.004	−0.101	0.904	0.841–0.972	0.006	−0.074	0.929	0.837–1.031	0.167

Note: B = unstandardized logistic regression coefficient; RRR = relative risk ratio; Class 1 = low decision regret group (reference); Class 2 = high decision regret group; Class 3 = moderate decision regret group.

## Data Availability

The data presented in this study are available on request from the corresponding author due to privacy and ethical restrictions.

## Supplementary Materials

The following supporting information can be downloaded at: https://www.mdpi.com/article/10.3390/curroncol33100612/s1. Table S1: Parsimonious sensitivity analysis of factors associated with latent profile membership (multinomial logistic regression, reference = Class 1); Table S2: Average marginal effects (AMEs) of predictors on profile membership; Table S3: Comparison of main analysis and corrected three-step (R3STEP) multinomial logistic regression results for factors associated with latent profile membership; Table S4: Firth penalized logistic regression for factors associated with high and moderate regret (reference: low regret).
