# Supplementary material for: Latent Profiles of Decision Regret and Associated Factors Among Patients Undergoing Medium- and Long-Term Vascular Access Device Placement in Medical Oncology: A Cross-Sectional Study
Source: Curr Oncol. 2026 Oct 12;33(10):612. doi: 10.3390/curroncol33100612 (PMC13703146; doi:10.3390/curroncol33100612)
Supplement: Supplementary file 1 [file curroncol-33-00612-s001.zip › curroncol-4518791-supplementary.pdf]

Supplementary Table S1. Parsimonious sensitivity analysis of factors associated with latent profile membership (multinomial logistic regression, reference = Class 1).

Variable	Class 2 vs Class 1 RRR (95% CI)	P	Class 3 vs Class 1 RRR (95% CI)	P
DCS score	1.18 (1.09–1.29)	<0.001	1.13 (1.05–1.21)	<0.001
SDM-Q-9 score	0.83 (0.76–0.91)	<0.001	0.90 (0.84–0.96)	<0.001
Employment (unemployed vs retired)	4.09 (1.08–15.51)	0.039	2.51 (0.90–7.01)	0.079
Employment (employed vs retired)	1.10 (0.14–8.77)	0.928	2.79 (0.90–8.66)	0.076

Note: RRR = relative risk ratio; Class 1 = low decision regret; Class 2 = high decision regret; Class 3 = moderate decision regret. Reference category for employment status was "retired".

Supplementary Table S2. Average marginal effects (AMEs) of predictors on profile membership

Predictor	Contrast	Class 1 (Low regret) AME (95% CI)	Class 2 (High regret) AME (95% CI)	Class 3 (Moderate regret) AME (95% CI)
DCS score	Per 10-point increase	-0.124 (-0.194, -0.054)***	0.053 (0.009, 0.096)*	0.071 (-0.0003, 0.143)
SDM-Q-9 score	Per 10-point increase	0.067 (0.032, 0.103)***	-0.042 (-0.068, -0.017)**	-0.025 (-0.064, 0.014)
Employment	Unemployed vs Employed	-0.011 (-0.096, 0.074)	0.066 (-0.006, 0.138)	-0.055 (-0.155, 0.045)
Retired vs Employed	Retired vs Employed	0.067 (-0.015, 0.149)	0.024 (-0.048, 0.096)	-0.091 (-0.189, 0.007)

Note: AMEs are expressed on the absolute probability scale. DCS = Decisional Conflict Scale; SDM-Q-9 = 9-item Shared Decision Making Questionnaire. *P < 0.05, **P < 0.01, ***P < 0.001. AMEs for DCS and SDM-Q-9 are reported per 10-point increase.

Supplementary Table S3. Comparison of main analysis and corrected three-step (R3STEP) multinomial logistic regression results for factors associated with latent profile membership.

Variable (reference)	Main analysis RRR (95% CI)	*P*	Corrected RRR (95% CI)	*P*
**Class 2 (high regret) vs Class 1 (low regret)**				
Rural (vs urban)	0.194 (0.046–0.815)	0.025	0.056 (0.004–0.829)	<0.001
Education: junior high school or below (vs bachelor's or above)	0.051 (0.002–1.359)	0.076	0.000*	<0.001
Education: senior high school/technical secondary school	0.021 (0.001–0.728)	0.033	0.000*	<0.001
Education: junior college	0.322 (0.011–9.618)	0.513	0.014 (0.000–3.291)*	<0.001
Employment: employed (vs retired)	2.579 (0.235–28.350)	0.439	2.318 (0.053–102.218)	0.768
Employment: unemployed/not working	30.546 (3.236–288.306)	0.003	1790.762 (4.990–642618.682)	0.739

Income: <3000 RMB (vs >8000 RMB)	1.795 (0.074–43.455)	0.719	NE (quasi-separation)	—
Income: 3000–5000 RMB	0.448 (0.017–12.141)	0.633	NE (quasi-separation)	—
Income: 5000–8000 RMB	1.364 (0.056–33.170)	0.849	NE (quasi-separation)	—
Device: PICC (vs midline catheter)	0.764 (0.041–14.261)	0.857	0.065 (0.001–6.360)*	<0.001
Device: implanted port	0.139 (0.007–2.945)	0.205	0.013 (0.000–1.619)*	<0.001
Family involvement in decision-making: yes (vs no)	0.551 (0.121–2.511)	0.441	0.468 (0.048–4.587)	0.329
Decision-related education: yes (vs no)	0.098 (0.017–0.570)	0.01	0.097 (0.014–0.675)	<0.001
DCS (per 1-point increase)	1.209 (1.092–1.340)	<0.001	1.304 (1.044–1.629)	0.04
SDM-Q-9 (per 1-point increase)	0.840 (0.746–0.946)	0.004	0.640 (0.426–0.962)	0.007
**Class 3 (moderate regret) vs Class 1 (low regret)**				
Rural (vs urban)	0.433 (0.122–1.532)	0.194	0.367 (0.090–1.495)	0.016
Education: junior high school or below (vs bachelor's or above)	0.787 (0.080–7.702)	0.837	0.326 (0.007–14.309)	0.284
Education: senior high school/technical secondary school	0.544 (0.063–4.726)	0.581	0.303 (0.008–10.839)	0.207
Education: junior college	0.649 (0.052–8.158)	0.738	0.440 (0.008–25.010)	0.537
Employment: employed (vs retired)	3.057 (0.827–11.302)	0.094	5.068 (0.806–31.877)	0.392
Employment: unemployed/not working	4.867 (1.255–18.874)	0.022	9.168 (1.895–44.353)	0.268
Income: <3000 RMB (vs >8000 RMB)	1.010 (0.135–7.584)	0.992	1.467 (0.206–10.457)	0.751
Income: 3000–5000 RMB	0.688 (0.099–4.789)	0.706	0.852 (0.113–6.407)	0.866
Income: 5000–8000 RMB	1.418 (0.206–9.772)	0.723	1.105 (0.100–12.266)	0.938
Device: PICC (vs midline catheter)	2.857 (0.227–35.899)	0.416	3.282 (0.355–30.375)	0.54
Device: implanted port	0.933 (0.073–11.903)	0.958	0.705 (0.077–6.482)	0.711
Family involvement in decision-making: yes (vs no)	0.333 (0.119–0.931)	0.036	0.307 (0.094–1.000)	<0.001
Decision-related education: yes (vs no)	0.140 (0.049–0.404)	<0.001	0.085 (0.027–0.263)	<0.001
DCS (per 1-point increase)	1.128 (1.043–1.220)	0.003	1.151 (1.053–1.258)	0.004
SDM-Q-9 (per 1-point increase)	0.904 (0.841–0.972)	0.006	0.880 (0.807–0.960)	0.002

Note: RRR = relative risk ratio; R3STEP = corrected three-step approach. The main analysis used multinomial logistic regression with most likely class membership as the dependent variable. Corrected RRRs were estimated using the R3STEP procedure in Mplus 8.3 to adjust for classification uncertainty. — = reference category or not reported; NE = not estimable due to quasi-separation. *Estimates are extreme and unreliable due to quasi-separation and the small size of the high-regret group (n = 28).

Supplementary Table S4. Firth penalized logistic regression for factors associated with high and moderate regret (reference: low regret).

Contrast	Variable	B	OR (95% CI)	*P*
Class 2 vs Class 1	DCS	0.149	1.161 (1.053-1.310)	0.002
	SDM-Q-9	−0.114	0.892 (0.803-0.983)	0.021
	Family involvement (yes vs no)	−1.041	0.353 (0.068-1.849)	0.214
	Education (high vs low)	−0.131	0.877 (0.148-4.786)	0.880
	Unemployed (vs employed/retired)	1.198	3.314(0.755-17.772)	0.114
Class 3 vs Class 1	DCS	0.126	1.134 (1.060-1.223)	<0.001
	SDM-Q-9	−0.082	0.921 (0.861-0.982)	0.012
	Family involvement (yes vs no)	−1.287	0.276 (0.108-0.702)	0.007
	Education (high vs low)	0.010	1.010 (0.378-2.699)	0.984
	Unemployed (vs employed/retired)	0.420	1.522 (0.593-3.913)	0.380

Note: Firth penalized logistic regression was used to address small-sample bias and quasi-separation. Class 2 vs Class 1 (n = 267) and Class 3 vs Class 1 (n = 281) were fitted separately. OR = odds ratio; CI = confidence interval; DCS = Decisional Conflict Scale; SDM-Q-9 = 9-item Shared Decision Making Questionnaire. The reference category for education was "junior high school or below"; for employment, the reference was "employed or retired."
